# Autoantibodies in Systemic Lupus Erythematosus: Diagnostic and Pathogenic Insights

**DOI:** 10.3390/jcm14165714

**Published:** 2025-08-12

**Authors:** Eleni Pagkopoulou, Charalampos Loutradis, Maria Papaioannou, Maria Daoudaki, Maria Stangou, Theodoros Dimitroulas

**Affiliations:** 1 Fourth Department of Internal Medicine, Hippokration Hospital, Aristotle University of Thessaloniki, 54642 Thessaloniki, Greece; elenipag4684@gmail.com (E.P.);; 2 First Department of Urology, G. Gennimatas Hospital, Aristotle University of Thessaloniki, 54635 Thessaloniki, Greece; mpapaioannou@auth.gr; 3 Laboratory of Biological Chemistry, School of Medicine, Aristotle University of Thessaloniki, 54124 Thessaloniki, Greece; daoudaki@auth.gr; 4 1st Department of Nephrology, Hippokration Hospital, School of Medicine, Aristotle University of Thessaloniki, 54642 Thessaloniki, Greece

**Keywords:** systemic lupus erythematosus, autoantibodies, lupus nephritis, targeted therapies

## Abstract

Systemic lupus erythematosus (SLE) is a chronic autoimmune disease characterized by widespread immune dysregulation and the production of autoantibodies targeting nuclear, cytoplasmic, and cell surface antigens. These autoantibodies are central to disease pathogenesis, contribute to immune complex formation and organ damage, and serve as essential diagnostic and prognostic markers. Their detection supports disease classification, guides clinical decision-making, and offers insight into disease activity and therapeutic response. Traditional markers such as anti-nuclear antibodies (ANA), anti-dsDNA, and anti-Sm antibodies remain diagnostic cornerstones, but growing attention is given to anti-C1q, anti-nucleosome antibodies (ANuA), anti-ribosomal P, antiphospholipid, and anti-cytokine antibodies due to their associations with specific disease phenotypes and activity. These markers may reflect disease activity, specific organ involvement, or predict flares. The mechanisms underlying their persistence include B cell tolerance failure and long-lived plasma cell activity. The aim of this review is to summarize current knowledge on the major autoantibodies in SLE, appraise available detection methods, highlight their clinical utility and limitations and present evidence on the association between antibodies and disease phenotypes.

## 1. Introduction

Systemic lupus erythematosus (SLE) is an autoimmune disorder with a highly variable clinical phenotype and course [1]. It affects multiple organs and systems, including the skin, joints, kidneys, nervous system, blood cells, and serous membranes among others. SLE predominantly affects women of childbearing age, with a 9:1 female-to-male ratio [2]. Geographic, ethnic, and socioeconomic factors diversify the epidemiologic profile of SLE, with higher incidence and severity observed among individuals of African, Hispanic, and Asian descent, as well as among populations with reduced access to healthcare [3,4].

A hallmark of SLE is the presence of a broad spectrum of autoantibodies directed against nuclear, cytoplasmic, and cell surface antigens [5,6]. These autoantibodies constitute a central role in the pathophysiology of the disease, contributing to immune complex formation, complement activation, and tissue inflammation [7,8]. The detection of specific autoantibodies is a prerequisite for the diagnosis of SLE, reflected in the inclusion of immunologic criteria in the 2019 EULAR/ACR classification criteria [9]. Beyond their diagnostic value, autoantibodies also provide prognostic insights, helping stratify patients based on risk of organ involvement, disease activity, and long-term outcomes [9]. Previous evidence suggests a strong association between specific autoantibodies and distinct clinical manifestations [10,11,12].

An understanding of immunopathology, diagnostic performance, and the clinical significance of the key autoantibodies is essential for the effective management of SLE. This review aims to provide an in-depth discussion of the immunological basis of autoantibodies production, the diagnostic and prognostic value of specific autoantibodies, and the evolution of emerging targets for precision medicine in SLE.

## 2. Pathophysiology of Autoantibodies in SLE

**Figure 1:**
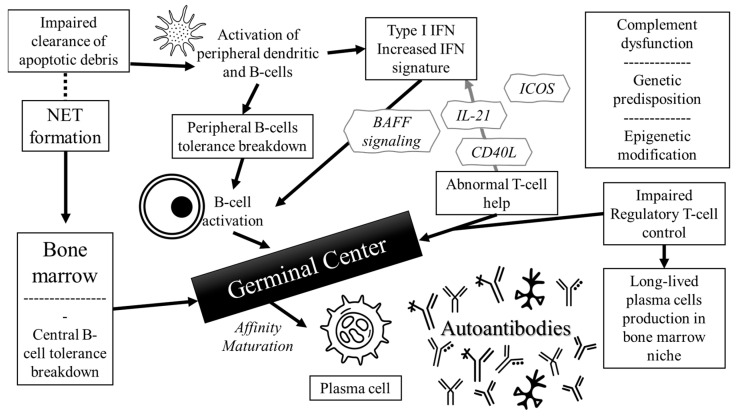
Pathophysiologic mechanism of antibody production in SLE. Key events include defective apoptotic debris clearance, neutrophil extracellular trap (NET) formation, and dysregulated type I interferon signaling, which stimulate plasmacytoid dendritic cells and autoreactive B cells via TLRs. B cell tolerance breakdown, excess BAFF signaling, and abnormal T cell help (CD40L, IL-21, ICOS) drive germinal center responses, affinity maturation, and plasma cell formation. Long-lived plasma cells produce high-affinity autoantibodies, perpetuating immune complex formation and chronic inflammation. The cycle is sustained by genetic susceptibility, complement dysfunction, and impaired regulatory T cell control.

The mechanistic background of antibody production in SLE is presented in Figure 1. The pathophysiology of autoantibody production includes defects in apoptotic clearance, dysregulated type I interferon (IFN) signaling, B cell tolerance breakdown, and abnormal T cell help [1]. Additional contributors include neutrophil extracellular trap (NET) formation, complement pathway dysfunction, genetic susceptibility, and epigenetic modifications [1]. These mechanisms sustain a chronic autoimmune background and the persistence of pathogenic autoantibodies. Autoantibody production results from complex disruptions in both innate and adaptive immunity. Central to this pathogenesis is the breakdown of B cell tolerance, both in the bone marrow and in peripheral tissues (central and peripheral tolerance), leading to the survival and activation of autoreactive B cells [13,14]. These B cells, when activated, undergo somatic hypermutation and affinity maturation in germinal centers, generating high-affinity, class-switched autoantibodies [15,16].

An early event in SLE pathogenesis is impaired clearance of the apoptotic debris, which allows intracellular autoantigens, such as nucleosomes, histones, and ribonucleoproteins, to persist extracellularly and be recognized as immunogens [16,17]. These nuclear components can form immune complexes that are internalized by plasmacytoid dendritic cells (pDCs) and B cells via Fc and Toll-like receptors (TLR), particularly TLR7 and TLR9 [18]. Activation of these receptors stimulates IFN production, especially IFN-α [19]. Results from seminal studies indicate that the “interferon signature”, a set of IFN-regulated genes, is abnormally upregulated in SLE patients and is associated with disease activity and autoantibody levels [20,21].

B cells in SLE exhibit multiple abnormalities, including increased expression of co-stimulatory molecules (CD40, CD80), increased survival signaling through the B cell-activating factor (BAFF), and resistance to apoptosis [22,23,24]. These factors contribute to the expansion of autoreactive clones and differentiation into plasma cells that produce pathogenic autoantibodies [25]. Long-lived plasma cells localize in survival niches within the bone marrow and spleen [26]. Moreover, T cells provide abnormal stimulation of the B cells through enhanced expression of CD40L, interleukin-21 (IL-21), and inducible T cell costimulator (ICOS), supporting germinal center reactions and the generation of memory B cells and plasma cells [27]. Dysregulated follicular helper T cells and defective regulatory T cells further exacerbate autoantibody production [28,29].

Sex-based immune differences may help explain why SLE is more common in females than males [30,31]. The X chromosome harbors multiple immune-related genes, including TLR7 and CD40L, which may escape X-inactivation and may be overexpressed in females [32,33,34]. This gene dosage effect can enhance B cell activation and IFN signaling [35]. Moreover, estrogens modulate immune responses by promoting B cell survival, increasing BAFF expression, and skewing T cell responses toward a Th2 phenotype, all of which favor autoantibody production and maintenance [36,37]. These mechanisms partly explain the striking female predominance and contribute to disease onset and flare patterns during periods of hormonal fluctuation, such as puberty or pregnancy.

## 3. Clinical Significance of Key Autoantibodies

**Figure 2:**
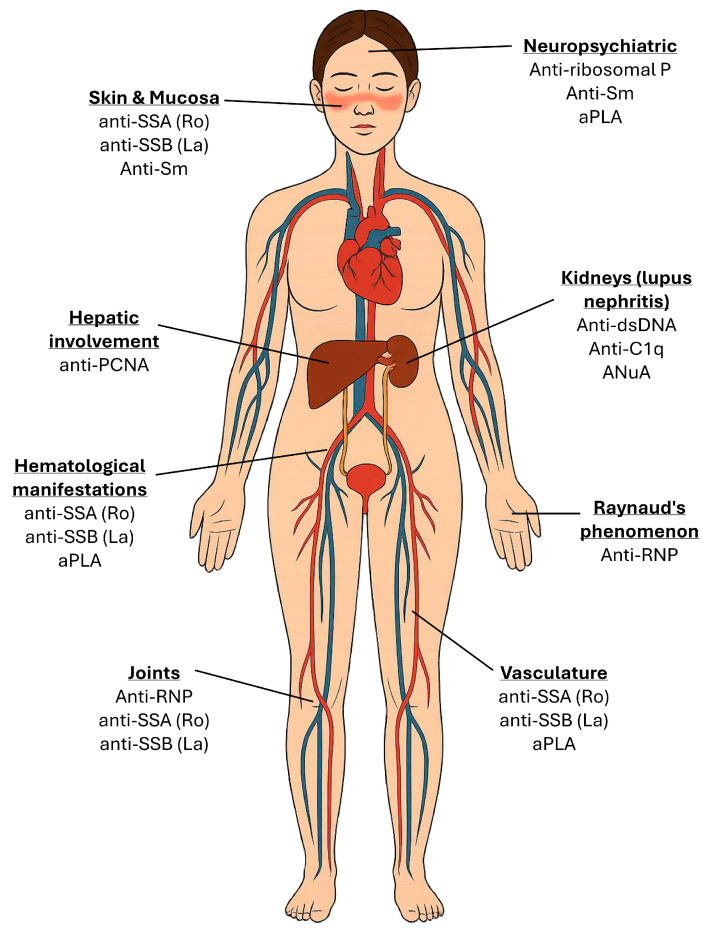
Major SLE-related organ manifestations and their corresponding autoantibody associations.

The serological spectrum of the existing autoantibodies in SLE represent a hallmark of the disease and provides critical insights into both the diagnosis and the disease heterogeneity. Importantly, these autoantibodies do not merely serve as serological biomarkers but also contribute to the underlying immunopathogenesis, with implications for disease monitoring, organ involvement, and prognosis [1,2]. The main antibodies encountered in SLE are presented in Table 1, while the major SLE-related organ manifestations and their corresponding autoantibody associations are presented in Figure 2.

Antinuclear antibodies (ANA) are the most sensitive but the least specific autoantibodies in SLE, present in >95% of cases and in a large proportion of the general population [38,39]. They typically precede the disease onset and are detected by indirect immunofluorescence assay (IIFA) on HEp-2 cells [40]. While ANA positivity is the entry criterion in the 2019 EULAR/ACR classification system (titer ≥ 1:80), their diagnostic utility is limited by their presence in other connective tissue diseases or in some healthy individuals, particularly at low levels [9]. Patterns such as homogenous, speckled, nucleolar, or centromere may offer clues to underlying autoantibody subsets and clinical associations [41]. ANA in SLE are broadly categorized into anti-DNA/nucleosome antibodies and anti-extractable nuclear antigen (ENA) antibodies, targeting DNA- or RNA-associated protein complexes, respectively [42].

**Table 1:** Clinical significance of key autoantibodies in SLE.

Autoantibody	Prevalence	Associated Clinical Features	Mechanism of Action
ANA	>95%	Screening; entry criterion in 2019 EULAR/ACR; non-specific	Bind nuclear antigens, activate innate immunity via Fc and TLR signaling [43].
Anti-dsDNA	50–70%	Lupus nephritis; flares; complement consumption	Bind double-stranded DNA forming nephritogenic immune complexes, activate complement, and deposit in glomeruli, triggering inflammation and tissue damage [44].
Anti-Sm	25–30%	Highly specific for SLE; systemic disease; NPSLE	Target snRNPs interfering with RNA splicing; form immune complexes that activate dendritic cells via TLRs, promoting type I IFN production and systemic autoimmunity; contribute to neurotoxicity via CNS penetration [45].
Anti-RNP	~40%	Raynaud’s; arthritis; overlap syndromes	Bind U1-RNP forming immune complexes that activate plasmacytoid dendritic cells via TLR7, enhancing type I IFN production [46].
Anti-Ro/SSA	30–40%	Cutaneous lupus; photosensitivity; neonatal lupus; hematologic involvement	Target Ro52/Ro60 ribonucleoproteins, forming immune complexes that activate dendritic cells via TLRs; cross placenta; bind cardiac tissue [47].
Anti-La/SSB	10–15%	Cutaneous lupus; neonatal lupus; hematologic involvement	Bind RNA-associated proteins forming immune complexes that activate Toll-like receptors and type I interferon pathways; mediate immune dysregulation [47].
Anti-ribosomal P	10–20%	Psychosis; depression; neuropsychiatric lupus	Target ribosomal P proteins; potential CNS penetration, disrupt neuronal function, and trigger neuroinflammation via cytokine release and immune complex formation [48].
ANuA	70–90%	Early SLE; lupus nephritis	Bind nucleosome complexes; promote immune complex formation, activate complement, and mediate glomerular deposition and glomerular inflammation [49].
Anti-histone	~30%	Drug-induced lupus	Bind to histone proteins within chromatin forming immune complexes; activate complement [50].
Anti-C1q	15–45%	Lupus nephritis	Bind the collagen-like region of C1q, impair apoptotic cell clearance, activate complement, and promote immune complex-mediated inflammation [51].
aPLA (LA, aCL, anti-β2GPI)	30–40%	Thrombosis; pregnancy loss; antiphospholipid syndrome	Bind phospholipid-bound proteins; activate endothelial cells, platelets, and complement; activate procoagulant and autoimmune mechanisms [52].

aCL: anti-cardiolipin antibody; ANA: antinuclear antibody; ANuA: anti-nucleosome antibody; Anti-C1q: anti-C1q complement antibody; Anti-dsDNA: anti-double stranded DNA antibody; Anti-La/SSB: anti-La/Sjögren’s Syndrome Type B antibody; Anti-histone: Anti-histone antibody; aPLA: antiphospholipid antibodies; Anti-RNP: anti-ribonucleoprotein antibody; Anti-Ro/SSA: anti-Ro/Sjögren’s Syndrome Type A antibody; Anti-Sm: anti-Smith antibody; Anti-ribosomal P: anti-Ribosomal P protein antibody; anti-β2GPI: anti-β2 glycoprotein I antibody; LA: lupus anticoagulant; snRNPs: small nuclear ribonucleoproteins.

Anti-double-stranded DNA (anti-dsDNA) antibodies are a type of antibodies against double-stranded DNA and are highly specific for SLE being present in 50–70% of patients [53]. Their presence is frequently associated with active lupus nephritis (LN), and their levels also correlate strongly with renal flares and complement consumption [54,55]. These antibodies form immune complexes that deposit in the mesangium, subendothelial, or subepithelial spaces near the glomerular basement membrane, triggering an inflammatory response, contributing directly to the pathogenesis of LN [56]. Their levels can be highly variable in parallel with SLE activity, with higher levels during flares and lower levels or even absence after appropriate immunosuppressive therapy [57].

Anti-RNA binding protein (anti-RBP) autoantibodies, present in 50% of SLE patients, comprise distinct subtypes such as anti-Smith (anti-Sm), anti-ribonucleoprotein (anti-RNP), anti-Ro/SSA, and anti-La/SSB, each targeting specific RNA-protein complexes and linked to characteristic clinical features [58]. The anti-Sm antibodies are highly specific but have about 20% sensitivity, being detected in approximately 25–30% of patients [59]. They are directed against small nuclear ribonucleoproteins and are more frequent (>30%) in African and Asian populations [60]. Although not tightly linked with specific clinical manifestations, their presence is considered a criterion with high diagnostic specificity and is often associated with more severe systemic disease [61]. Evidence from literature suggests a possible association between anti-Sm antibodies and diffuse neuropsychiatric SLE, particularly acute confusional states, likely reflecting their transudation into the cerebrospinal fluid through a compromised blood–brain barrier and their potential neurotoxic effects on neuronal cells [62].

Anti-RNP antibodies are often co-expressed with anti-Sm and are associated with features of overlapping syndromes, particularly mixed connective tissue disease [42,63]. In SLE, they are detectable in 25–47% of patients and may correlate with Raynaud’s phenomenon, arthritis, and reduced renal involvement [39]. Anti-RNP antibodies are considered products of long-lived plasma cells, and their levels tend to remain stable over time, resembling antibody responses to vaccines or persistent pathogens; consequently, they are not typically regarded as markers of disease flares in SLE. Their presence supports the notion of overlapping autoimmunity and broad B cell activation [64].

The Anti-Sjogren’s syndrome A (anti-SSA) and B (anti-SSB) are also known as Anti-Ro and anti-La accordingly. The anti-SSA are encountered in 30–40% and the anti-SSB in 10–15% of SLE patients [65]. These antibodies are more common in patients with cutaneous manifestations and vasculitis (palpable purpura), photosensitivity, and hematologic involvement (anemia, leukopenia, and thrombocytopenia), but these associations are not consistent in all studies [66,67,68]. Their relevance extends beyond adult disease, as maternal anti-Ro antibodies are strongly linked to neonatal lupus and congenital heart block [69]. These antibodies may be present years before the clinical onset of SLE and are associated with late SLE onset, underscoring their role in early disease pathogenesis [70].

Antiphospholipid antibodies (aPLA), including lupus anticoagulant, anti-cardiolipin, and anti-β2 glycoprotein I antibodies, define the immunological background of the antiphospholipid syndrome (APS), which can occur alone or in association with SLE [71]. They are linked to arterial and venous thrombosis, recurrent pregnancy loss, pre-eclampsia, thrombocytopenia, anemia, livedo reticularis, valvular heart disease and neurologic symptoms such as migraine or cognitive dysfunction [72,73,74,75]. Persistent aPLA positivity, particularly at high titers and with triple-positive profiles, is associated with increased risk of thrombosis and may require specific therapeutic approaches, with vitamin k antagonists or heparin/low molecular weight heparin being the preferred treatment for secondary APS thrombosis prophylaxis [71,76]. The use of the direct oral anticoagulants remains controversial; current guidance suggests their use for non–triple-positive patients under specific clinical circumstances [77].

Anti-ribosomal P antibodies target ribosomal P proteins [78]. Although results from earlier studies suggested possible association with neuropsychiatric manifestations of SLE, including psychosis and depression, recent large-scale prospective studies failed to confirm a consistent association. A meta-analysis of 22 studies reported a modest association between anti-ribosomal P antibodies and neuropsychiatric SLE, though significant heterogeneity and inconsistent findings, including in the SLICC cohort, limit their diagnostic utility [79]. Their prevalence in SLE varies between 10 and 47%, depending on factors like immunoassay type, ethnicity, region, cohort design, and disease onset age [79]. Although they are not part of current classification criteria, they may provide diagnostic support in patients with otherwise unexplained central nervous system involvement [48]. The ANuA recognize complexes of histones and DNA and are considered highly sensitive for SLE, being positive in 70–90% of patients [80]. ANuA may precede the appearance of anti-dsDNA antibodies and they have been associated with increased disease activity and renal involvement [81]. They promote immune complex formation and deposition on glomerular basement membranes, thereby initiating inflammatory cascades and increasing capillary permeability [16,82]. Anti-histone antibodies are found in over 75% of patients with drug-induced lupus and in 30% of those with idiopathic SLE [83]. Their presence must be interpreted within the clinical context, particularly in patients exposed to drugs known to induce lupus-like syndromes, such as isoniazid and penicillamine [84]. Anti-C1q antibodies have emerged as potential biomarkers of LN, correlating with histologic activity and renal flares. They appear in 15–45% of SLE patients and may offer additive predictive value alongside anti-dsDNA and complement levels [85]. C1q plays a role in apoptotic cell clearance; hence, its targeting by autoantibodies reflects a central defect in immune tolerance [86].

In addition to the commonly evaluated autoantibodies, several others may be detected in patients with SLE, particularly in the context of overlap syndromes. Anti-proliferating cell nuclear antigen (anti-PCNA) antibodies are rare, with their prevalence ranging between 1 and 10%, but may be associated with active disease and hepatic involvement [87]. Antibodies to Ku protein (anti-Ku) and anti-polymyositis/scleroderma antibodies (anti-PM-Scl) are often seen in overlap syndromes with polymyositis or systemic sclerosis and may indicate a mixed connective tissue phenotype [88,89]. Anti-Scl-70 (topoisomerase I) and anti-centromere antibodies are more characteristic of systemic sclerosis, but can occasionally be found in SLE patients, particularly in those with sclerodermatous features or Raynaud’s phenomenon [90]. Anti-histidyl-tRNA synthetase (anti-Jo-1) and other anti-synthetase antibodies are typical of anti-synthetase syndrome, but may signal an overlap with myositis presenting with interstitial lung disease or proximal muscle weakness [91]. Mi-2 beta antigen (anti-Mi-2) and anti-fibrillarin antibodies are also associated with dermatomyositis and systemic sclerosis, respectively [92]. Autoantibodies targeting cytokines such as type I and II interferons [93], BAFF [94], and IP-10 [95] have also been reported in SLE and may reflect disease activity and predict flares. While not part of routine lupus serology, identification of the latter antibodies can aid in diagnosing complex or atypical presentations, guide screening for systemic features, and support tailored therapeutic strategies.

## 4. Autoantibodies in Clinical Diagnosis

The diagnostic framework of SLE relies heavily on the detection of autoantibodies, which serve as objective biomarkers and are an integral part of the classification criteria [9]. Advances in serological technologies have enabled earlier recognition of disease and facilitated subclassification based on autoantibody profiles. The most widely employed diagnostic algorithm begins with ANA screening, which remains a prerequisite for fulfilling contemporary classification systems, despite their limited specificity. However, their mere presence is insufficient for diagnosis, necessitating subsequent autoantibody characterization through antigen-specific assays [9].

In this context, the detection of additional antibodies assumes particular importance. The specificity of anti-dsDNA for SLE is high, and their presence often satisfies one of the key immunological domains in diagnostic scoring systems [9]. Their diagnostic value is augmented when considered alongside complement levels (C3 and C4), as hypocomplementemia and elevated anti-dsDNA levels form a reliable surrogate marker for disease exacerbation [55]. Anti-Sm antibodies, despite low sensitivity, are among the few autoantibodies with near-absolute diagnostic specificity and are incorporated into current classification criteria [45]. Their co-expression with anti-RNP antibodies raises suspicion for overlap syndromes, although their diagnostic specificity for SLE remains unmatched [96]. Anti-Ro/SSA and anti-La/SSB antibodies, though not SLE-specific, contribute to diagnostic precision by identifying patients with prominent mucocutaneous involvement, photosensitivity, or hematological abnormalities [65]. Maternal anti-Ro/SSA antibodies are clinically significant even in asymptomatic pregnant women, as they are associated with the risk of congenital heart block in the fetus [97]. The detection of antiphospholipid antibodies in the setting of SLE carries both diagnostic and prognostic implications [71]. In patients with SLE, screening for aPLA is essential, especially prior to initiating estrogen therapy or during pregnancy planning, and when positive in patients with thrombosis or pregnancy morbidity, they establish the diagnosis of APS [98]. As autoantibody profiles may vary in clinical relevance, their interpretation should be made within the disease context, integrated with the disease course and supported by standardized, context-driven diagnostic strategies.

The detection and characterization of autoantibodies in SLE rely on various immunological techniques, each with distinct advantages and limitations [40]. Indirect immunofluorescence assay (IIFA) on HEp-2 cells remains the gold standard for ANA screening, due to its high sensitivity and ability to reveal nuclear staining patterns that may hint at specific autoantibody profiles [99]. However, IIFA interpretation is inherently subjective and dependent on operator expertise [100]. Enzyme-linked immunosorbent assays (ELISA) are commonly used for the detection of specific autoantibodies such as anti-dsDNA, anti-Sm, and anti-RNP, offering quantitative results and suitability for routine clinical use to evaluate disease activity and treatment response [101]. More recently, multiplex immunoassays and bead-based technologies have enabled the simultaneous measurement of multiple autoantibodies in a single reaction, improving the efficiency and expanding the diagnostic process [102]. Nevertheless, differences in assay sensitivity, antigen source, and cut-off values between laboratories introduce variability, complicating inter-assay comparisons and longitudinal monitoring. This lack of methodological standardization underscores the importance of validating test results across platforms and interpreting serological findings in close correlation with clinical presentation.

## 5. Lupus Nephritis and Autoantibodies

Among the organ-specific complications of SLE, LN is one of the most common and potentially severe manifestations, characterized by immune complex deposition in the glomeruli and subsequent inflammatory injury [103]. Approximately 10–30% of patients have LN when diagnosed with SLE, while 30–50% may present renal involvement within the first five years of the disease course [104].

The pathogenesis of LN is complex and involves interactions among autoantibodies, complement activation, and immune dysregulation. The anti-dsDNA antibodies are prominently associated with renal involvement [1]. Their presence correlates strongly with the occurrence and severity of nephritis, particularly in class III and IV proliferative LN [105]. These antibodies form pathogenic immune complexes with nuclear antigens that are deposited in the glomerular basement membrane and mesangium, triggering complement activation and inflammatory cascades [106]. Clinical studies demonstrate that rising anti-dsDNA levels frequently precede renal flares, and sustained elevation often portends poor renal prognosis [107,108]. Moreover, anti-C1q antibodies, by targeting a key component of the classical complement pathway, impair apoptotic debris clearance and promote immune complex deposition in renal tissue [109]. Their elevated levels are closely associated with active lupus nephritis and may help predict histological severity on biopsy [110]. The ANuA, though less commonly tested, have been linked to LN and may serve as an early marker of renal involvement, especially in those with anti-dsDNA absence [81]. Noticeably in LN, CD8^+^ T cells, IL-17–producing T cells, macrophages, and B cells infiltrate the kidney, promoting inflammation and tissue damage [111]. CD8^+^ T cells invade the tubular epithelium, IL-17 drives inflammation, and B cells form germinal center–like structures producing anti-vimentin antibodies [112,113].

The diagnostic workup for LN integrates clinical evaluation with laboratory biomarkers, i.e., presence of hematuria, proteinuria, hypertension and renal function estimation [114,115]. Quantification of autoantibodies (anti-dsDNA, anti-C1q), complement levels (C3, C4), and the evaluation of the urinary sediment supports diagnosis and guides therapeutic decisions. However, the renal biopsy remains the gold standard for histologic classification and prognostication, particularly in patients with new-onset or worsening proteinuria or renal function decline [114,115]. Per-protocol kidney biopsies, performed at predefined timepoints regardless of clinical status, may help assess histologic response, guide maintenance therapy duration, and improve long-term prognostication in LN [116]. From a prognostic point-of-view, patients with persistently high autoantibody levels and low complement levels have an increased risk of recurrent renal flares and chronic kidney disease development [117]. In a retrospective cohort study in 921 SLE patients, anti-dsDNA and anti-nucleosome antibodies were associated with increased risk for multiple LN flares, particularly in patients with delayed LN onset after SLE diagnosis [118].

## 6. Therapeutic Implications and Emerging Targets

The central role of autoantibodies in the pathogenesis and progression of SLE shape the basis upon which therapeutic interventions are implemented. These interventions essentially include the reduction in their production or mechanism of actions. The management of SLE and LN includes immunosuppressive agents such as glucocorticosteroids, hydroxychloroquine, azathioprine cyclophosphamide, and mycophenolate mofetil to control antibody-mediated inflammation [119]. While effective in attenuating inflammation and preventing flares, these therapies lack specificity and are often associated with substantial toxicity [9,114,115]. Targeted therapies aiming to reduce pathogenic autoantibody production (e.g., anti-CD20 monoclonal antibodies, anti-BAFF agents, type I IFN receptor blockade and IFN signaling inhibition) are increasingly used in refractory or relapsing LN, further highlighting the central role of humoral autoimmunity in disease pathogenesis [119,120]. The novel and emerging treatments targeting autoantibodies in SLE are summarized in Table 2.

Among the most thoroughly investigated targeted therapies is the use of B cell-directed agents. Rituximab, a chimeric monoclonal antibody targeting CD20, which depletes circulating B lymphocytes was one of the first targeted treatments tested in refractory cases of SLE, particularly those with renal or neuropsychiatric involvement [121]. In the EXPLORER trial of 257 extrarenal SLE patients, rituximab failed to improve primary or secondary outcomes over placebo, though subgroup benefit was noted in African American and Hispanic patients [122]. In the LUNAR trial including 144 patients with class III/IV LN, rituximab plus mycophenolate mofetil (MMF) and steroids improved serologic markers and reduced cyclophosphamide rescue (0% vs. 11%) but did not significantly increase renal response at 52 weeks compared to placebo (56.9% vs. 45.8%, *p* = 0.18), with a comparable safety profile [123]. In another study Rituximab achieved an 85% response rate in 35 refractory neuropsychiatric SLE cases, often enabling steroid tapering, relapse occurred in 45% and infections in 29%, supporting cautious off-label use [124]. Importantly, large randomized controlled trials on Rituximab in SLE are missing.

Belimumab, a monoclonal antibody targeting BAFF, was the first biologic agent approved by Food and Drug Administration (FDA) for SLE. By inhibiting BAFF belimumab decreases the survival of autoreactive B cells, reduces disease activity and flares in both non-renal SLE and LN. The efficacy of belimumab was evaluated in a phase III trial of 819 SLE patients with ≥6 Safety of Estrogens in Lupus Erythematosus National Assessment (SELENA) and SLE Disease Activity Index (SLEDAI). At 10 mg/kg, belimumab improved the SLE Responder Index (SRI, 43.2% vs. 33.5%, *p* = 0.017) at week 52 compared to placebo with a favorable safety profile [125]. In another phase III trial in 865 SLE patients with SELENA-SLEDAI ≥ 6, 10 mg/kg belimumab improved SRI response at week 52 [58% vs. 44%, Odds ratio (OR) 1.83; *p* = 0.0006), reduced SELENA-SLEDAI and British Isles Lupus Assessment Group (BILAG) flares, with similar adverse event rates to placebo [126]. In the BLISS-LN trial including adults with biopsy-proven LN, 10 mg/kg belimumab and standard therapy significantly improved primary renal response at 104 weeks (43% vs. 32%; *p* = 0.03), complete renal response (30% vs. 20%; *p* = 0.02) and reduced the risk of renal events or death (HR 0.51; *p* = 0.001), with a favorable safety profile [127]. In refractory SLE, belimumab after rituximab reduced anti-dsDNA levels by 70%, lowered severe flares (HR 0.27; *p* = 0.033), and suppressed B cell repopulation without added safety concerns [128]. Belimumab has also demonstrated substantial efficacy in pediatric patients with SLE [129].

Anifrolumab is a humanized IgG1k monoclonal antibody that binds to subunit 1 of the type-1 IFN receptor (IFNAR) and inhibits the formation of the IFN/IFNAR complex and subsequent gene transcription [130]. In the phase IIb MUSE trial, 300 mg anifrolumab improved SRI at week 24 (34.3% vs. 17.6%, *p* = 0.014), especially in IFN-high patients (36.0% vs. 13.2%, *p* = 0.004), with sustained week-52 benefits in SRI, BICLA, and major response compared to placebo [131]. In a 3-year open-label extension of the MUSE trial, anifrolumab showed durable SLEDAI and serologic improvement, stable organ damage, quality of life scores, and acceptable safety (≥1 adverse events: 69.7%; discontinuation: 6.9%) compared to placebo [132]. In the phase 3 TULIP-1 trial including 457 patients, anifrolumab (300 mg/4 weeks) failed to meet the primary endpoint (SRI reduction at week 52: 36% vs. 40%, *p* = 0.41), but results indicated benefits in steroid reduction (41% vs. 32%), Cutaneous LE Disease Area and Severity Index (CLASI) improvement (42% vs. 25%), and BICLA response (37% vs. 27%), with a comparable safety profile to placebo [133]. In the phase 3 TULIP-2 trial (n = 362), anifrolumab (300 mg/4 weeks) significantly increased BICLA response at week 52 vs. placebo (47.8% vs. 31.5%, *p* = 0.001), especially in patients with high interferon signatures, and reduced steroid use and skin disease severity, though flare rates and joint counts were unaffected [134]. Following the results of the above trials Anifrolumab is the second biologic therapy approved by the FDA for patients with SLE without active LN or severe active central nervous involvement [135].

Obinutuzumab is an anti-CD20 monoclonal antibody with enhanced Fc effector function which induces programmed cell death in B cells and was originally developed for the treatment of B cell malignancies [136]. In lupus-prone hCD20 MRL/lpr mice, obinutuzumab resulted in greater B cell depletion, clinical efficacy, LN remission, and reduced anti-RNA antibodies, and CD4^+^ T cell activation compared to rituximab [137]. In the phase 2 NOBILITY trial involving 125 patients with active biopsy-proven LN (classes III, IV with or without class V), obinutuzumab added to MMF and steroids improved complete renal response in LN at week 104 (41% vs. 23%, *p* = 0.026), alongside better serologic and eGFR outcomes, with no increase in serious adverse events [138]. In a small study including nine SLE patients with secondary nonresponse to rituximab, obinutuzumab (2000 mg) led to significant reductions in SLEDAI and BILAG scores, improved C3 and anti-dsDNA levels, and enabled the tapering of steroids in most patients [139].

The effects of rontalizumab, an anti-IFN-α monoclonal antibody neutralizing all 12 IFN-α subtypes, in patients with moderate-to-severe SLE were evaluated in the ROSE trial. The results indicated similar BILAG and SRI response rates between rontalizumab and placebo; on the other hand, in a post hoc analysis of an ISM-low subgroup, rontalizumab was associated with improved SRI responses, reduced flare rates (HR 0.61, *p* = 0.004), and decreased steroid use [140]. In addition, Sifalimumab, another human IgG1κ antibody neutralizing most IFN-α subtypes, demonstrated promising efficacy in a phase IIb trial (n = 431), with higher SRI response rates at week 52 compared to placebo (58–60% vs. 45%) and improvements across multiple disease activity indices. Although well-tolerated, herpes zoster infections were more frequent with sifalimumab [141]. Despite promising phase II results, clinical development was halted for both agents due to limited efficacy in larger trials.

Targeting T cell co-stimulation pathways has emerged as a therapeutic strategy in SLE. Abatacept, a fusion protein combining cytotoxic T-lymphocyte-associated protein 4 (CTLA-4) with the Fc portion of IgG1, modulates CD80/CD86:CD28 signaling and is approved for rheumatoid arthritis [142]. In SLE, it has been assessed in multiple trials with contradictory results. Although a phase II trial failed to meet its primary endpoint in non-life-threatening diseases, some improvements were observed in polyarthritis [143]. In LN, abatacept did not significantly improve complete renal response at 24 weeks (33% vs. 31%); however, 50% of responders maintained remission after discontinuing immunosuppressants [144]. Other agents targeting co-stimulation include lulizumab pegol (anti-CD28), which failed to meet the primary endpoint of BICLA response rates at week 24, as well its secondary endpoints, including CLASI [145]. The anti-CD40L dapirolizumab pegol showed promising immunologic and gene expression changes in phase I but did not meet efficacy endpoints in phase II [146]. Additional agents such as BI 655,064 (anti-CD40), VAY736, CFZ533, and ruplizumab also failed to achieve significant benefit or were prematurely terminated [147,148]. Despite setbacks, targeting co-stimulatory pathways remains a rational and evolving therapeutic strategy.

Janus kinases (JAKs) and signal transducer and activator of transcription (STAT) proteins mediate cytokine and growth factor signaling across immune cells [149]. The JAK/STAT pathway is crucial for immune tolerance, and its dysregulation contributes to autoimmune diseases, including SLE [150,151]. Both in vitro and in vivo studies support its role in SLE pathogenesis [152], and STAT gene polymorphisms have been linked to increased risk of SLE and LN [153]. Baricitinib, a JAK 1/2 inhibitor, demonstrated adequate efficacy in cutaneous and articular SLE in a phase II trial [154]; in murine LN models, it reduced renal inflammation and restored podocyte structure [155]. Tofacitinib, another JAK 1/3 inhibitor, showed safety and improvement in cardiometabolic and immunologic parameters, including type I IFN signature, in a phase I study [156].

**Table 2:** Targeted Therapies in SLE against specific autoantibodies or their production.

Agent	Target	Mechanism	Indications	Clinical Key Findings
Rituximab	CD20+ B cells	Depletes mature B cells; reduces autoantibody production	Refractory SLE/LN/NPSLE	Similar to placebo effects in the EXPLORER [122] and the LUNAR [123] trials. Off-label use supported in refractory NPSLE (85% response, 45% relapse), reduces anti-dsDNA, steroid-sparing [124].
Belimumab	BAFF inhibition	Inhibits B cell survival	Non-renal and renal SLE	Improved SRI-4 and renal response in BLISS-52/76 and BLISS-LN trials; FDA-approved; steroid-sparing [125,126,127]. Significant improvement in refractory SLE and efficacy in pediatric SLE [128,129].
Anifrolumab	IFNAR1	Blocks type I IFN receptor, inhibiting IFN signaling	Non-renal SLE	MUSE: increased SRI-4 (34.3% vs. 17.6%, *p* = 0.014), benefit in IFN-high [131]. TULIP-1: no SRI-4 benefit, signal in BICLA and CLASI [133]. TULIP-2: ↑BICLA (47.8% vs. 31.5%, *p* = 0.001), steroid-sparing, skin benefits [134].
Obinutuzumab	CD20+ B cells	Type II anti-CD20 antibody; induces enhanced B cell apoptosis	Refractory LN post-rituximab	NOBILITY: improved CRR (41% vs. 23%, *p* = 0.026) with improvements from baseline in C3, C4 anti-dsDNA and eGFR (adjusted mean difference, 9.7 mL/min/1.73 m^2^ (95% CI 1.7–18), *p* = 0.017) [138].
Rontalizumab	IFN-α	Neutralizes all 12 IFN-α subtypes	SLE (low ISG subset)	ROSE: no overall benefit vs. placebo; ISM-low subgroup: improved SRI, reduced flares (HR 0.61, *p* = 0.004), steroid-sparing [140].
Sifalimumab	IFN-α	Neutralizes most IFN-α subtypes	SLE	Phase IIb: improved SRI-4 at week 52 (58–60% vs. 45%), broad disease activity improvement, increased occurrence of herpes zoster infection [141].
Abatacept	CD80/CD86	Inhibits CD28 co-stimulation on T cells	SLE, LN	ACCESS: no improvement in CRR at 24 weeks (33% vs. 31%); 50% of abatacept responders sustained remission after stopping immunosuppressants [144].
Dapirolizumab	PEGylated anti-CD40L Fab	Inhibits T cell–B cell interaction via CD40–CD40L axis	SLE	Phase II: modest improvement in BICLA, SRI-4, and serologic markers vs. placebo; dose–response not met (*p* = 0.07) [146].
Baricitinib	JAK 1/2	Inhibits JAK-STAT signaling; reduces inflammatory cytokine signaling	Cutaneous/articular SLE	Improvement in arthritis/rash resolution at week 24 (67% vs. 53%, *p* = 0.041); preclinical data support renoprotection via JAK/STAT modulation [154,155].
Tofacitinib	JAK 1/3	Modulates type I IFN responses and T cell activation	Investigational	Phase I: improved HDL profile, vascular function, and IFN signature in SLE; benefits stronger in STAT4-risk carriers [156].

BICLA: BILAG-based composite lupus assessment; CD: cluster of differentiation; CI: confidence interval; CLASI: cutaneous lupus erythematosus disease area and severity index; CRR: complete renal response; dsDNA: double-stranded deoxyribonucleic acid; eGFR: estimated glomerular filtration rate; FDA: food and drug administration; HDL: high-density lipoprotein; HR: hazard ratio; IFN: interferon; IFNAR: interferon-α/β receptor; ISG: interferon-stimulated genes; ISM: interferon signature metric; JAK: Janus kinase; LN: lupus nephritis; NPSLE: neuropsychiatric systemic lupus erythematosus; PEG: polyethylene glycol; SLE: systemic lupus erythematosus; SRI-4: SLE Responder Index (≥4 point improvement); STAT: signal transducer and activator of transcription.

## 7. Challenges and Future Directions

Despite advances in SLE immunopathogenesis and targeted therapies, major challenges remain. Disease heterogeneity hampers early diagnosis, risk assessment, and treatment, since no single biomarker or therapy is effective for all phenotypes [157]. Existing autoantibody profiles in SLE can offer insights into the disease and its diagnosis, their correlation with disease activity is often inconsistent, but changes in these profiles tend to follow, clinical manifestations of disease flares [158]. Thus, their inability to reliably predict disease flares or long-term outcomes hampers individualized care and contributes to both overt- and under-treatment [159]. Additionally, the widespread use of non-specific immunosuppressants, although effective in acute settings, carries considerable long-term toxicity [9,114]. The definitions of remission and low disease activity state differ among studies and guidelines, and their application in routine clinical practice is not uniform [160].

Importantly, many patients exhibit clinical remission but persistent immunologic activity, e.g., elevated anti-dsDNA and hypocomplementemia [161]. The relevance of treating serologically active but clinically quiescent (SACQ) lupus remains controversial. In a recent *p* a pooled analysis of five phase III trials, clinical remission or lupus low disease activity state (LLADAS) with normal serology are associated with a lower risk for severe or renal flares compared to those patients who presented serologically active remission (persistent anti-dsDNA positivity or low C3/C4 levels), possibly indicating deeper states of disease control in the former patients [162]. However, these associations are modest, and there is no consensus on initiating or escalating therapy solely based on serologic activity. Current recommendations emphasize individualized risk assessment, and further studies are needed to determine whether targeting serologic activity in the absence of clinical symptoms improves long-term outcomes [115].

A future challenge includes leveraging multi-omics technologies, which may lead to more precise and personalized SLE care [163]. These platforms may help identify novel biomarkers capable of predicting flares, therapeutic response, and organ-specific involvement. Advances in transcriptomics (e.g., IFN gene signatures), miRNA profiling (e.g., miR-146a, miR-155), proteomic panels (e.g., S100A9), and metabolomics could lead to clinically actionable stratification tools, though validation, standardization, and cost-effectiveness remain barriers to widespread use [164,165,166,167]. Meanwhile, emerging therapeutic avenues, including agents targeting microbiome, metabolic checkpoints, and epigenetic regulators, are under investigation [168]. Addressing long-standing challenges such as equitable care access, underrepresentation in clinical trials, and the psychosocial impact of SLE will require coordinated research, community engagement, and patient-centered care models.

## 8. Conclusions

Autoantibodies continue to play a central role in the diagnosis, pathogenesis, and clinical stratification of SLE, offering insights into disease heterogeneity and prognosis. Despite advances in serological technologies and targeted therapies, their utility is limited by variable specificity, poor correlation with disease activity in some contexts, and delayed serologic responses to flares or treatment. Anti-dsDNA and anti-C1q antibodies retain significant predictive value in LN, while the presence of anti-Sm, anti-Ro, and anti-ribosomal P antibodies assists in the delineation of neuropsychiatric, cutaneous, or hematologic phenotypes. Emerging autoantibody targets and molecular signatures, including IFN-regulated gene profiles, may advance precision medicine but require further validation. Regarding treatment, biologic agents directed against B cells, BAFF, and type I interferon pathways have reshaped the treatment landscape and demonstrated efficacy in reducing disease activity and organ damage, yet a significant proportion of patients remains refractory. Integrating traditional serology with multi-omics platforms may improve risk stratification, enable earlier intervention, and guide individualized care. Continued investment in mechanistic research, equitable trial design, and standardized implementation of validated biomarkers will be essential to refine treatment paradigms and improve long-term outcomes in SLE.

## Data Availability

This article is a review and does not include original data.

## Abbreviations

ANA	Anti-nuclear antibodies
aCL	Anti-cardiolipin antibody
ANuA	Anti-nucleosome antibodies
anti-dsDNA	Anti-double-stranded DNA antibody
anti-C1q	Anti-C1q antibody
anti-Jo-1	Anti-histidyl-tRNA synthetase
anti-Ku	Antibodies to Ku protein
anti-La	Anti-La/SSB antibody
anti-PCNA	Anti-proliferating cell nuclear antigen
anti-PM-Scl	Anti-polymyositis/scleroderma antibodies
aPLA	Antiphospholipid antibodies
APS	Antiphospholipid syndrome
anti-RBP	Anti-RNA binding protein
anti-RNP	Anti-ribonucleoprotein
anti-Ro,	Anti-Ro/SSA antibody
anti-Sm	Anti-Smith antibody
anti-SSA	Anti-Sjogren’s syndrome A
anti-SSB	Anti-Sjogren’s syndrome B
BAFF	B cell-activating factor
BILAG	British Isles Lupus Assessment Group
CD	Cluster of differentiation
CLASI	Cutaneous Lupus Erythematosus Disease Area and Severity Index
CTLA-4	Cytotoxic T-lymphocyte-associated protein 4
ELISA	Enzyme-linked immunosorbent assays
ENA	Extractable nuclear antigen
HR	Hazard ratio
ICOS	Inducible T cell costimulator
IFN	Type I interferon
IFNAR	Interferon-alpha receptor
IIFA	Indirect immunofluorescence assay
IL-21	Interleukin-21
JAKs	Janus kinases
LLADAS	Lupus low disease activity state
MMF	Mycophenolate mofetil
NET	Neutrophil extracellular trap
pDCs	Plasmacytoid dendritic cells
SELENA-SLEDAI	Safety of Estrogens in Lupus Erythematosus National Assessment-Systemic Lupus Erythematosus Disease Activity Index
SLE	Systemic lupus erythematosus
STAT	Signal transducer and activator of transcription
TLR	Toll-like receptors
